# Enhanced Biological Behavior of *In Vitro* Human Gingival Fibroblasts on Cold Plasma-Treated Zirconia

**DOI:** 10.1371/journal.pone.0140278

**Published:** 2015-10-13

**Authors:** Miao Zheng, Yang Yang, Xiao-Qiang Liu, Ming-Yue Liu, Xiao-Fei Zhang, Xin Wang, He-Ping Li, Jian-Guo Tan

**Affiliations:** 1 Department of Prosthodontics, Peking University School of Stomatology, 22# Zhongguancun South Street, Haidian District, Beijing, 100081, People’s Republic of China; 2 Department of Engineering Physics, Tsinghua University, Beijing 100084, People’s Republic of China; 3 College of Mechanical Engineering, North China University of Science and Technology, Tangshan 063009, People’s Republic of China; University Paul Sabatier, FRANCE

## Abstract

**Objective:**

To evaluate whether atmospheric-pressure dielectric-barrier-discharge plasma treatment of zirconia enhances its biocompatibility with human gingival fibroblasts.

**Materials and Methods:**

The zirconia disks were divided into four groups and treated using helium atmospheric-pressure dielectric-barrier-discharge plasmas for 30, 60 or 90 s or left untreated. The surface morphology, wettability and chemical elements were analyzed. Fibroblasts density, morphology, morphometry and attachment-related genes expression were measured at different time points from 3 to 72 h.

**Results:**

After plasma treatment, the surface morphology and roughness remained the same, while the contact angle decreased from 78.31° to 43.71°, and the surface C/O ratio decreased from 3.17 to 0.89. The surficial areas and perimeters of HGFs were increased two-fold in the treated groups at 3 h. Fibroblasts density increased on treated disks at all time points, especially the ones treated for 60 s. Attachment-related genes in the groups treated for 30 and 60 s were significantly higher at 3 and 24 h.

**Conclusion:**

The helium atmospheric-pressure dielectric-barrier-discharge plasma treatment enhances the biological behavior of fibroblasts on zirconia by increasing the expression of attachment-related genes within 24 h and promoting the cell density during longer culture times. Wettability of zirconia, an important physicochemical property, has a vital influence on the cell behaviors.

## Introduction

The long-term success of dental implants depends on the integrity of osseointegration, the health of the epithelium and the quality of attachment of the connective tissue to the abutment surface. The transmucosal area constitutes a barrier between the oral environment and peri-implant bone, and thus, forms an effective biological soft tissue seal, which protects the implant by resisting challenges from bacterial irritants [1,2]. Human gingival fibroblasts (HGFs) are major collagen fiber-producing cells located in peri-implant connective tissue [3], and there are more HGFs in the connective tissue immediately next to the abutment surface [4]. This is why HGFs have been the subject of most *in vitro* studies [5,6,7].

Both materials type and surface properties of abutments affect the biological behavior of the nearby connective tissue and that of the HGFs. From the aspect of materials type of the implants and abutments, titanium is a traditional material used for both implants and abutments due to its remarkable mechanical properties and biocompatibility; while its dark color limits its use in the esthetic zone. Zirconia has been introduced in recent years as a promising material for implant abutment because of its good biocompatibility [8,9], desirable mechanical properties [10], low plaque affinity [11] and excellent esthetic outcomes [12]. On the other hand, among various properties of the materials, surface roughness and wettability are two vital factors of the surface properties which affect the biological behaviors of the cells at the materials interface. Previous studies emphasized that the smooth titanium surface was more suitable to the HGFs [13]. And the experiments on zirconia also gave the similar conclusion in [8] that the smooth zirconia with a roughness of 0.04 μm benefited the growth of HGFs. With respect to the influences of the surface wettability on the attachment and proliferation abilities of cells, previous studies indicated that the hydrophilic surfaces at a moderate level which were prepared by different methods, for example, using self-assembled monolayers of alkanethiols with different terminal groups, UV irradiation or by plasma treatment, were suitable for cell growth [14,15,16,17,18,19,20,21].

There are various methods to change surfaces wettability [22,23,24,25], but some of these methods, e.g., sand blasting [24], chemical modification [25] or UV light treatment [23] may change the surface topography or take a long treatment time, which may change the materials mechanical properties [10] or make it inconvenient for clinical usage. In recent studies, plasmas, as the fourth state of matter, play an essential role for the effective surface modifications of biomedical materials in dentistry [16,18,20,21,26,27]. These studies have already shown the ability of plasmas to enhance the surface wettability of zirconia efficiently [21,28], which could enhance the behavior of oral keratinocytes [21] and osteoblasts [29] without changing the surface morphology. While to our knowledge, there are few studies focusing on the HGFs behavior on the plasma-treated zirconia. So, the objective of this study was to enhance the bioactivity of zirconia abutment materials treated by a helium atmospheric-pressure dielectric-barrier-discharge (APDBD) plasma. In this study, the surface roughness and contact angle of the zirconia disks were measured before and after the helium APDBD plasma treatment. The biological behavior of the HGFs on a plasma treated zirconia specimens was analyzed *in vitro* for the first time, which includes the adhesion and proliferation ability and morphology of the HGFs at a cellular level; and the expression of the specific cell adhesion-related genes at a molecular level.

## Materials and Methods

### Ethics statement

HGFs were grown from biopsies obtained from a periodontally healthy human subject during periodontal surgery with the advance approval of the Institutional Review Board of Peking University School of Stomatology, Beijing, China (PKUSSIRB-2012060). A total of 10 participants participated in this study, and all signed written informed consent before the periodontal surgeries. The ethics committee approved this consent procedure.

### Preparation of zirconia disks

The zirconia disks (Zenostar, Wieland Dental, Pforzheim, Germany) were 20 mm in diameter and 2 mm in thickness. The crystallographic structure of zirconia was analyzed previously and the results suggested that it fitted the properties of zirconium yttrium oxide [23]. All specimens were grinded under water coolant using a nominally 60-μm grit size diamond cup wheel (25 mm diameter, Grish Hitech Co., Beijing, China) mounted on a grinding machine (AutoMet 300, Buehler, Waukegan, IL, USA). Grinding conditions were as follows: a 15 N single force was applied vertically on the disks using a head speed of 50 rpm and platen speed of 100 rpm, with a grinding time of 20 minutes. Immediately before surface treatment, the disks were washed ultrasonically in absolute ethanol and distilled water for 20 minutes, respectively. All disks were divided into four groups according to the plasma treatment time, in which three experimental groups were treated by the plasmas for 30, 60 or 90 s, while one control group with no plasma treatment for comparison. Right before the cellular and molecular experiments, all disks were rinsed with PBS three times.

### Plasma source for the materials surface modifications

**Fig 1:**
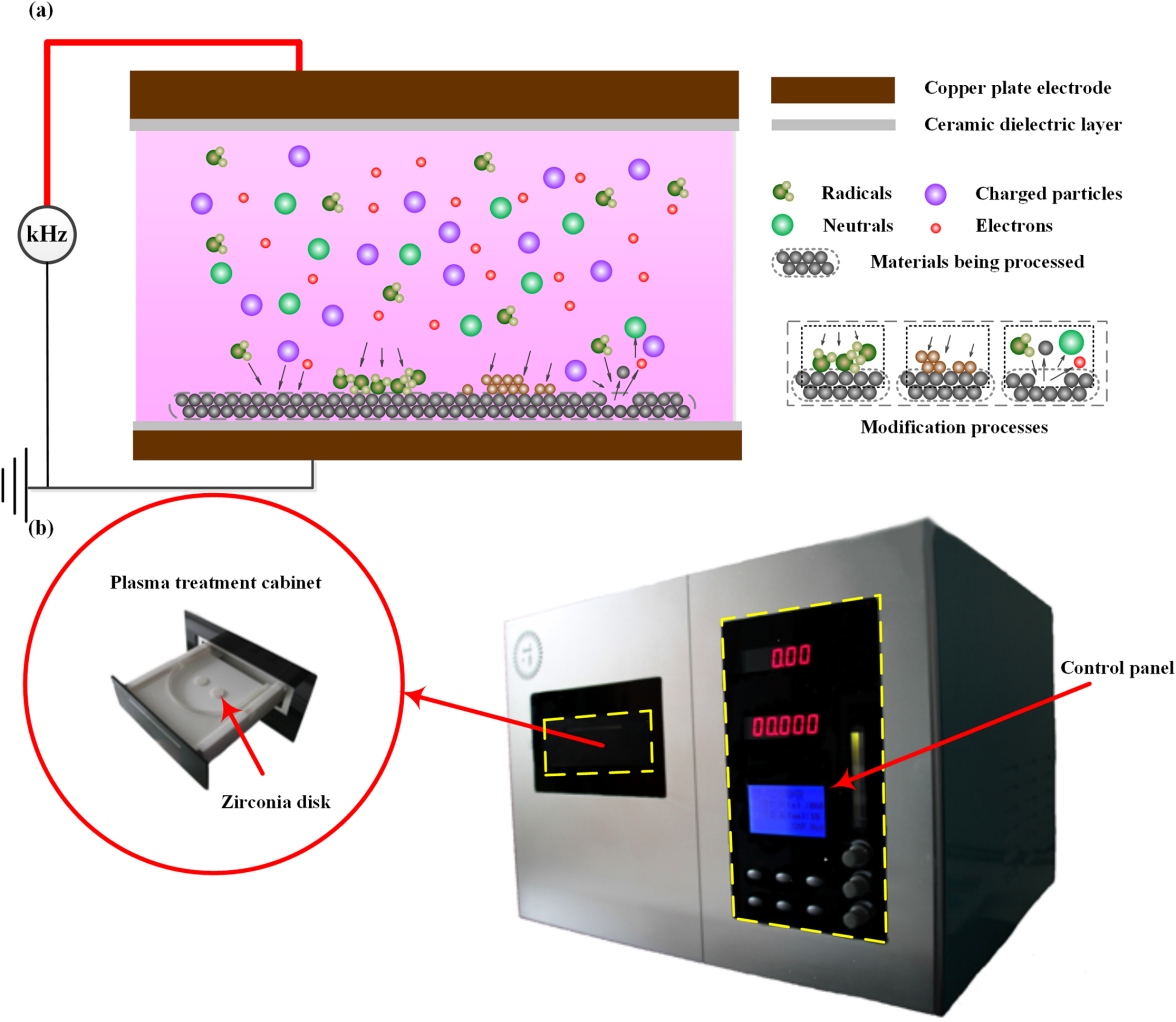
Schematic and picture of the APDBD plasma generator. Schematic of the APDBD plasma generator and plasma materials treatment processing (a), and the picture of the CAP Mod-I machine for the treatment of zirconia disks (b).

In this study, a helium atmospheric-pressure dielectric-barrier-discharge (APDBD) plasma source was employed for the surface modification of the zirconia disks. As shown in Fig 1(A), a high-frequency high-voltage power supply was applied to generate homogeneous and stable plasmas with the DBD generator which was composed of two copper plate electrodes, each of which was covered by a 2.0- mm-in-thickness ceramic dielectric layer. The materials being processed (e.g., zirconia disks in this study) were placed upon the lower ceramic plate in the discharge region which contained different types of chemically reactive species, such as electrons, ions, neutral radicals, etc. And just resulting from the existence of these reactive species, a variety of chemical and physical processes may take place during the plasma treatment process of biomedical materials, as schematically illustrated in Fig 1(A). Based on this direct plasma treatment method, a plasma materials processing machine, CAP Mod-I (Beijing Zhenrong Rongtong Communication Co., Ltd., Beijing, China), has been developed, as shown in Fig 1(B), which mainly includes the plasma treatment cabinet and the control panel. In this study, high-purity helium at a flow rate of 10.8 slpm was used as the plasma forming gas; and the uniform glow discharge was obtained at the fixed volume of 25.25 cm^3^ with the power density of 0.36–0.44 W/cm^3^, applied voltages of 4.64–4.72 kV at a driving frequency of 26 kHz. For avoiding the influence of surrounding air on the characteristics of the helium glow discharge plasmas since the discharge operates at an open environment, a helium blowing at a flow rate of 16.2 slpm before discharge was conducted so as to flush the air out of the discharge space between electrodes.

### Surface morphology and roughness

The surface morphology of all four groups of zirconia disks was examined using scanning electron microscopy (SEM) (S-4800, Hitachi, Tokyo, Japan). The surface roughness was characterized using an analyzer of roughness (Mitutoyo Surftest 401 Analyzer Series 200, Mitutoyo Corp., Minatoku, Japan). The arithmetical mean surface roughness (Ra in μm) was determined using a cut-off value of 0.8 mm and a measurement length of 4 mm. Each disk underwent five measurements at different locations, and the average value of five disks was used as the measured roughness for each group.

### Surface wettability

Surface wettability of each group was examined using a contact angle meter (SL200, USA Kino Industry, Norcross, GA,USA). Measurements were made at three different locations on each disk 3 s after application of a 1-μL H_2_O droplet. Three disks from each group were examined, and the average value coming from these three disks was used as the measured wettability for each group.

### XPS analysis

The quantitative mean atomic composition of zirconia in terms of carbon and oxygen contents was examined using X-ray photoelectron spectroscopy (XPS) (ESCALAB 250, ThermoFisher Scientific, Waltham, MA, USA). The survey spectra were measured (200 eV pass energy) and detailed spectra (30 eV pass energy) were obtained for the C 1s, Zr 3d and O 1s peaks. All spectra were normalized to the binding energy scale of the C1s peak (284.8 eV).

### Cell culture

HGFs were grown from biopsies obtained from a periodontally healthy human subject during periodontal surgery. The HGF medium was Dulbecco’s modified Eagle’s medium (Gibco BRL Co., Gaithersburg, MD, USA) supplemented with 10% fetal bovine serum (Gibco BRL Co.) and 1% antibiotic–anti- mycotic solution. The cells were placed in an incubator at 37°C in an atmosphere of 95% humidity and 5% carbon dioxide. Confluent cells were subcultured by trypsinization. The medium was replaced every 2 days. All experiments were performed using cells between the fourth and seventh passages.

### Cell adhesion and proliferation assays (CCK-8 analysis)

To evaluate the adhesion and proliferation of HGFs on the zirconia disk surfaces, cells were seeded at a density of 1.0×10^5^/zirconia disk in the 24-well culture plates (n = 5 for each group). Cells were allowed to adhere to the disks for half an hour before complete medium (1 mL/well) was added to the well. HGFs were allowed to grow for 3, 24, 48 or 72 h. The Cell Counting Kit-8 (CCK-8, Dojindo, Kyushu, Japan) was used to evaluate the cell adhesion and proliferation. When evaluated at each time point, cells were first washed with phosphate buffed saline three times before adding the CCK-8 solution (100 μL/mL of cell culture medium). After incubation at 37°C for 2 h, the optical density of the solution was measured using a spectrophotometer (ELX808, BioTek, Winooski, VT, USA) at a wavelength of 450 nm. All the experiments were repeated in triplicates.

### Scanning electron microscopic analysis

The scanning electron microscopy (SEM) (S-4800, Hitachi, Tokyo, Japan) was used to evaluate the morphology of the cells attached to the zirconia disks. In this study, 5×10^4^ or 1×10^4^ cells were seeded onto disks and incubated for 3 or 24 h, respectively, to observe the cell attachment and spreading. For conducting the SEM examination, specimens were fixed with 4% formaldehyde for 2 h at room temperature and rinsed three times in deionized water for 15 min; then, each specimen was dehydrated in an ascending ethanol series (ranging from 30 to 100% ethanol), three times for 10 min at 4°C. Finally these samples were sputter coated with gold palladium for 60 s at 60 mA (SCD050, Balzers, Liechtenstein).

### Confocal laser scanning microscopic analysis

Indirect immunofluorescence (IIF) using fluorescein isothiocyanate–phalloidin (actin filament green color,Sigma, St. Louis, MO, USA) and 4’6-diamidino-2-phenylindole (nuclei blue color,Roche, Basler, Switzerland) was performed for morphometric examination. Either 5×10^4^ or 1×10^4^ cells were seeded on disks and incubated for 3 or 24 h, respectively. The unattached cells were rinsed with sterile PBS. The attached cells were then fixed in 4% paraformaldehyde for 50 min and rinsed in PBS three times (5 min each). Thereafter, the samples were stained with fluorescein isothiocyanate–phalloidin. After extensive rinsing with PBS, a drop of Fluoroshield containing 4',6-diamidino-2-phenylindole was added, and coverslips were mounted onto the disks. Finally, the samples were observed using a confocal laser scanning microscopy (LSM710,Zeiss, Germany). The cell area and perimeter were evaluated using an image analyzer (Image J, version 2, NIH).

### Gene expression analysis

The relative gene expression of adhesion marker integrins (α5, β3) and fibronectin (FN) was assessed at the mRNA level using a real-time polymerase chain reaction (real-time PCR). The total mRNA from six samples per group after surface treatment was isolated using a TRIzol (Invitrogen, Grand Island, NY, USA) after 3, 24, 48 or 72 h of culturing, and then pooled. The RNA concentration was measured using a Biophotometer (Eppendorf, Hamburg, Germany). The total RNA (2 μg) was converted into cDNA using the RevertAid First Strand cDNA Synthesis Kit (Thermo Scientific, Waltham, MA, USA) according to protocol provided by the manufacturer. The real-time PCR reactions were performed using the 7500 real-time PCR System (Applied Biosystems, Grand Island, NY, USA) with the specific primers shown in Table 1, the Power SYBR Green PCR Master Mix (Applied Biosystems) and cDNA equivalent to 30 ng total mRNA. The relative mRNA expression of integrins (α5, β3) and FN was normalized to the housekeeping gene GADPH and was analyzed using the comparative ΔΔCT method.

**Table 1:** Primer sequences for the real-time polymerase chain reaction.

Gene	Sequences (5’-3’)	
	Forward	Reverse
Integrin α5	GGCAGCTATGGCGTCCCACTGTGG	GGCATCAGAGGTGGCTGGAGGCTT
Integrin β3	TGACGAAAATACCTGCAACCG	GCATCCTTGCCAGTGTCCTTAA
Fibronectin	CGGAGAGACAGGAGGAAATAGCCCT	TTGCTGCTTGCGGGGCTGTC
GAPDH	TGCACCACCAACTGCTTAGC	GGCATGGACTGTGGTCATGAG

### Statistical analysis

All the experiments were repeated three times in this study, and the averaged data were provided with the corresponding standard deviations. A one-way analysis of variance (ANOVA) with Tukey method was performed. A significance level of 0.05 was set in all statistical comparisons. All data analyses were performed using the SPSS 17.0 software (SPSS, IBM).

## Results

### Surface morphology and photo-generated hydrophilicity of zirconia

**Fig 2:**
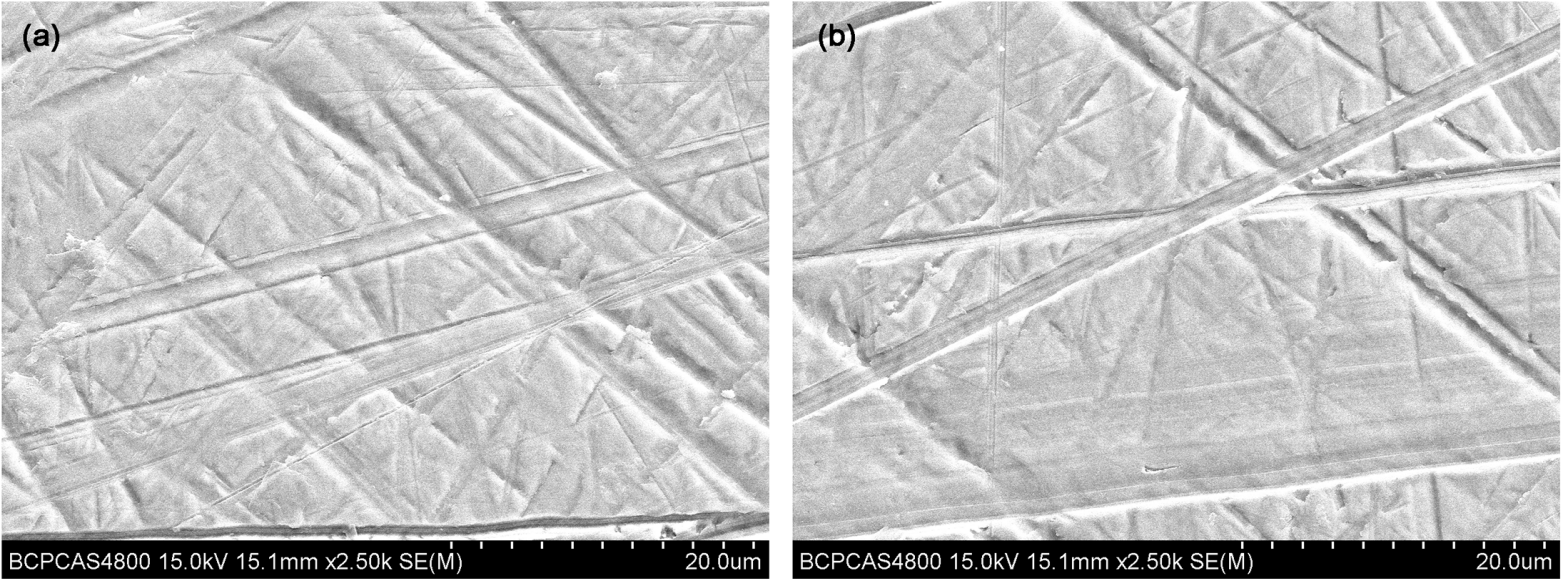
Surface topography observations of the zirconia disks before and after the plasma treatment. SEM images of the control (a) and zirconia treated by the helium APDBD plasma (b).

**Fig 3:**
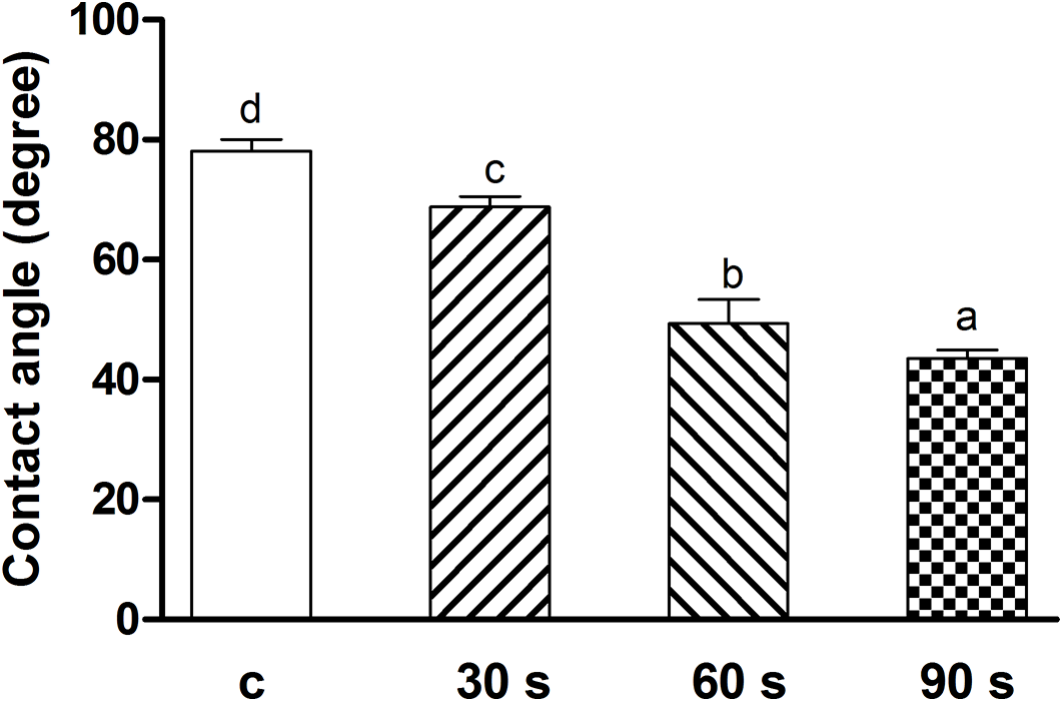
Surface contact angles of the control and plasma treatment surfaces. The surface contact angles of zirconia decreased significantly after the helium APDBD plasma treatment for 30, 60 or 90 s. Data are shown as means ± SD (n = 9). Identical letters indicate no significant difference for each value (*p* > 0.05).

The average roughness (Ra) of the zirconia disks from four groups was 0.05±0.01 μm and remained the same after the helium APDBD plasma treatment for 30, 60 or 90 s. The SEM images before and after plasma treatment are shown in Fig 2. It is seen that all the specimens have a relatively smooth morphology with some typical traces from the grinding process. After the cold plasma treatment, the contact angles of the surfaces decreased significantly from 78.31° to 43.71°, which resulted in a more hydrophilic surface (Fig 3).

### XPS analysis of zirconia

**Fig 4:**
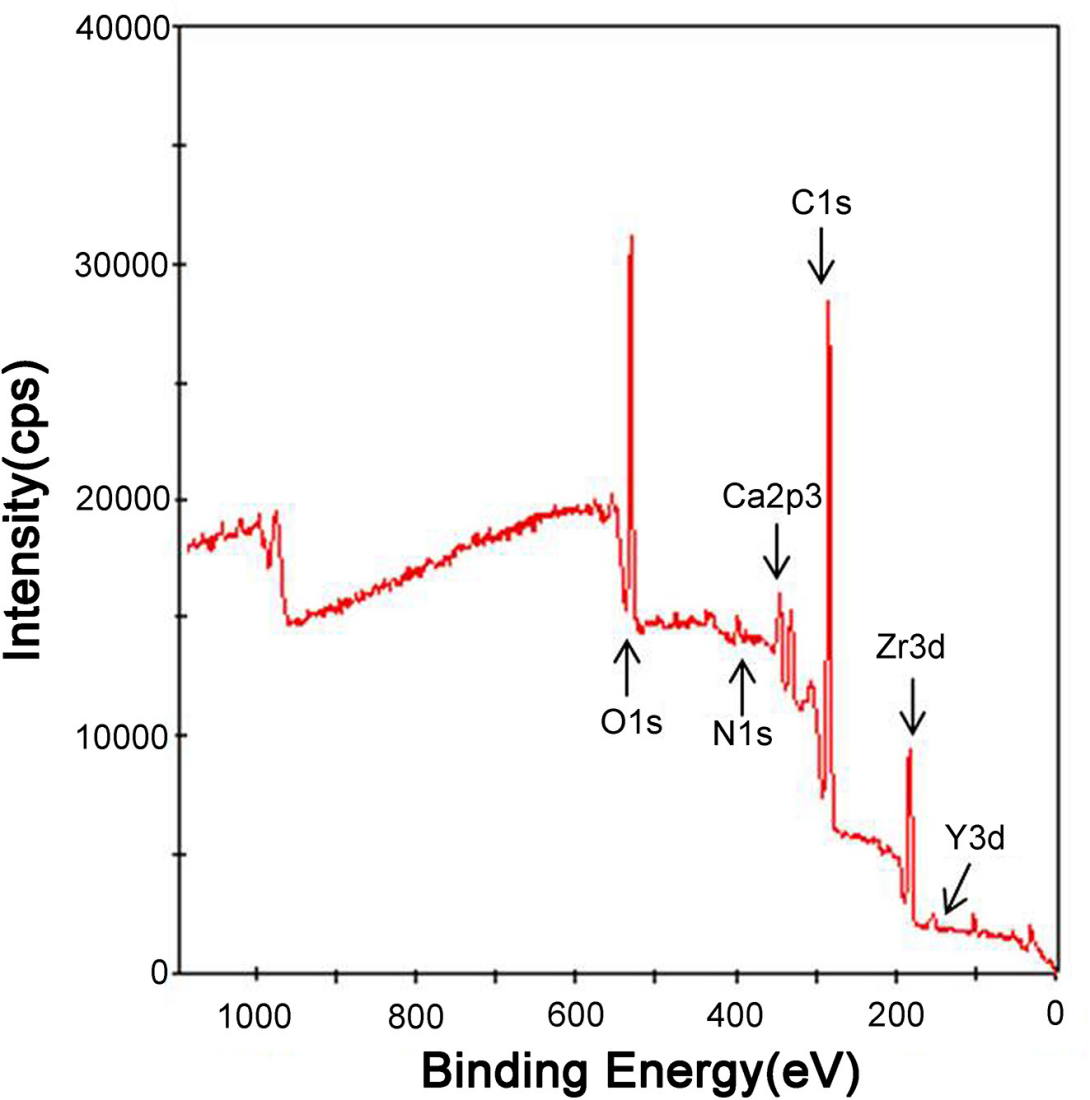
XPS spectrum of the zirconia disks.

**Fig 5:**
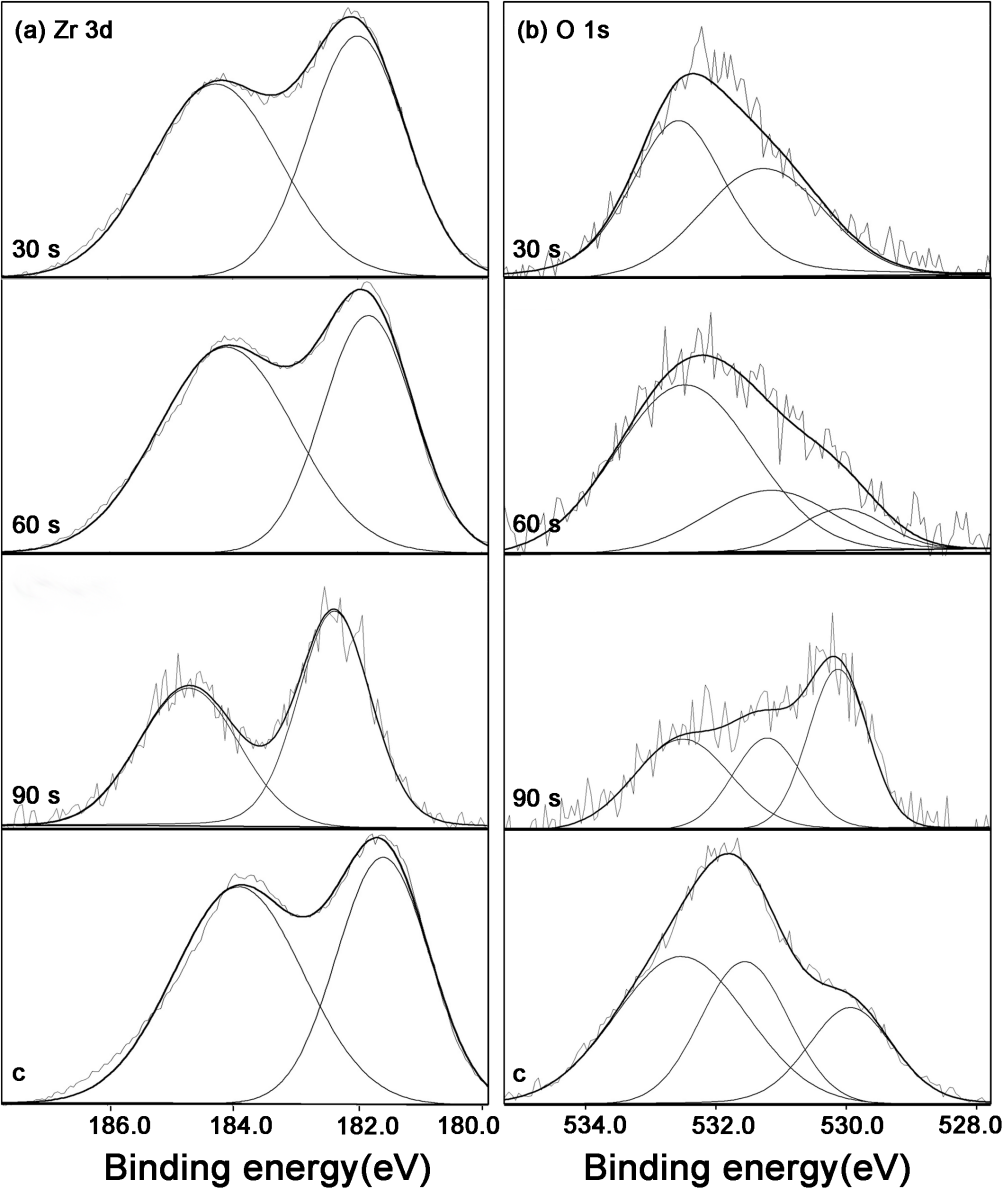
High-resolution XPS spectra of the control and plasma treatment surfaces.

The XPS analysis of zirconia specimens showed peaks of C 1s, O 1s, Ca 2p_3_, N 1s, Y 3d, and Zr 3d (Fig 4). After the helium APDBD plasma treatment, the atomic percentage of carbon (at %) on the outermost surface of the three groups decreased, as did the surface C/O ratio (Table 2). As shown in Fig 4, the Zr 3d spectra exhibit a pair of Zr 3d_5/2_ and Zr 3d_3/2_ peaks at 182.0 and 184.4 eV, corresponding to Zr 3d in ZrO_2_ [30]. The O 1s spectra of the oxidized Zr metal could be fitted with three symmetrical, mixed Gaussian-Lorentzian peak components: (1) Dissolved, BE = 530.1 ± 0.1 eV, (2) Oxide, BE = 531.2 ± 0.1 eV, (3) Hydroxide, BE = 532.5 ± 0.2 eV [31] (Fig 5). The relative area ratios of the three deconvoluted peaks are calculated and listed in Table 3. It is shown that the area percentage of hydroxide increased in the 30 and 60 s groups, while decreased in the 90 s group.

**Table 2:** Atomic percentage of C1s and O1s on four surfaces.

	C1s (at %)	O1s (at %)	C/O Ratio
30s	60.91	31.02	1.96
60s	56.33	31.6	1.78
90s	33.2	37.18	0.89
control	68.25	21.55	3.17

**Table 3:** Area ratios of the peaks obtained by decovoluting the XPS O 1s spectra for the plasma-treated zirconia surfaces and that of the control.

	Area ratios of the deconvoluted peaks in O 1s spectra		
	530.1 eV	531.2 eV	532.5 eV
30 s	1.67	42.43	55.90
60 s	10.44	22.44	67.12
90 s	38.81	25.60	35.59
c	21.86	30.59	47.55

### Influence of different surfaces on the attachment and proliferation of HGFs

**Fig 6:**
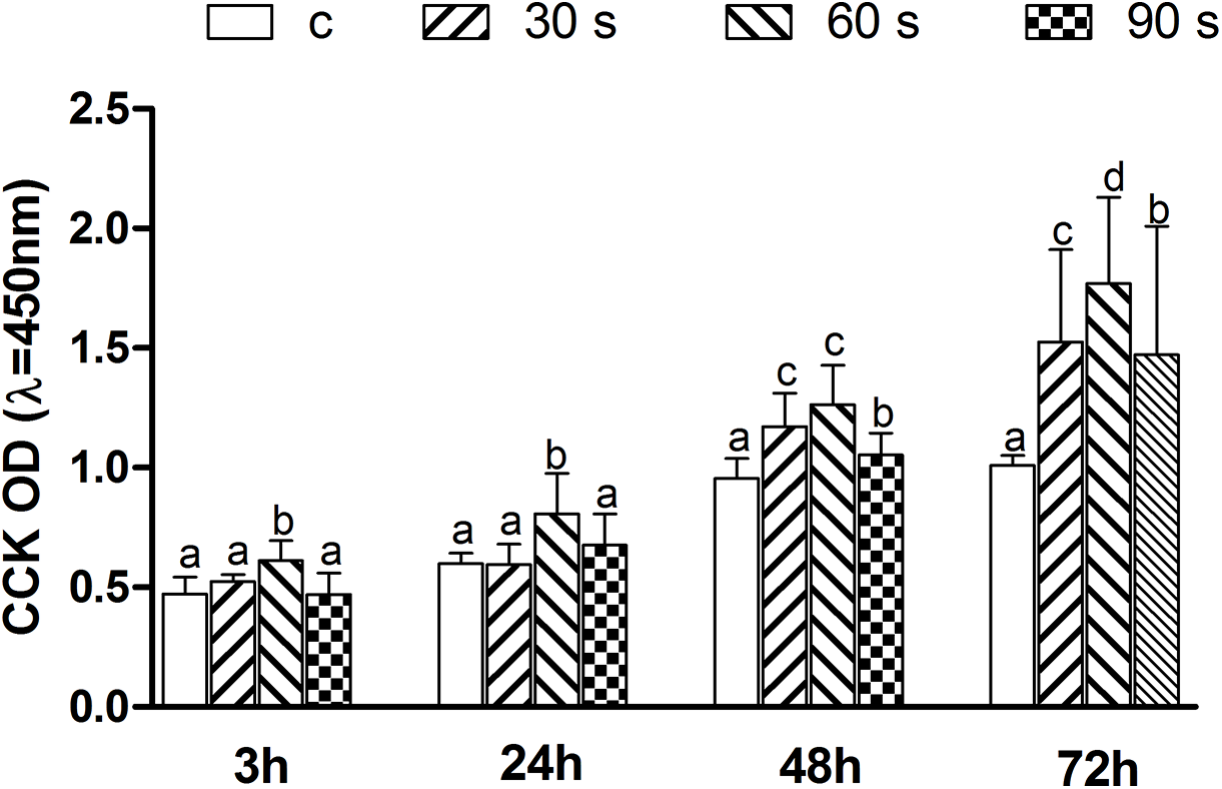
Quantitative measurements of the HGFs on the control and plasma treatment surfaces. Cell attachment after culturing for 3 and 24 h, and proliferation after culturing for 48 and 72 h. Data are shown as mean ± SD (n = 15). Identical letters indicate no significant difference between those values (*p* > 0.05).

The optical densities of the attached HGFs at 3, 24, 48 and 72 h of incubation were significantly higher for all the plasma treated zirconia surfaces compared with those of the control group at 48 and 72 h (*p*<0.05) (Fig 6). Among the three plasma treatment groups, the 60 s group had the largest number of attached cells at 3, 24 and 72 h (*p*<0.05), followed by the 30 and 90 s groups at 48 and 72 h (*p*<0.05). As incubation time increased, the advantage of the plasma treatment process became more significant.

### Influence of different surfaces on the morphology of HGFs

**Fig 7:**
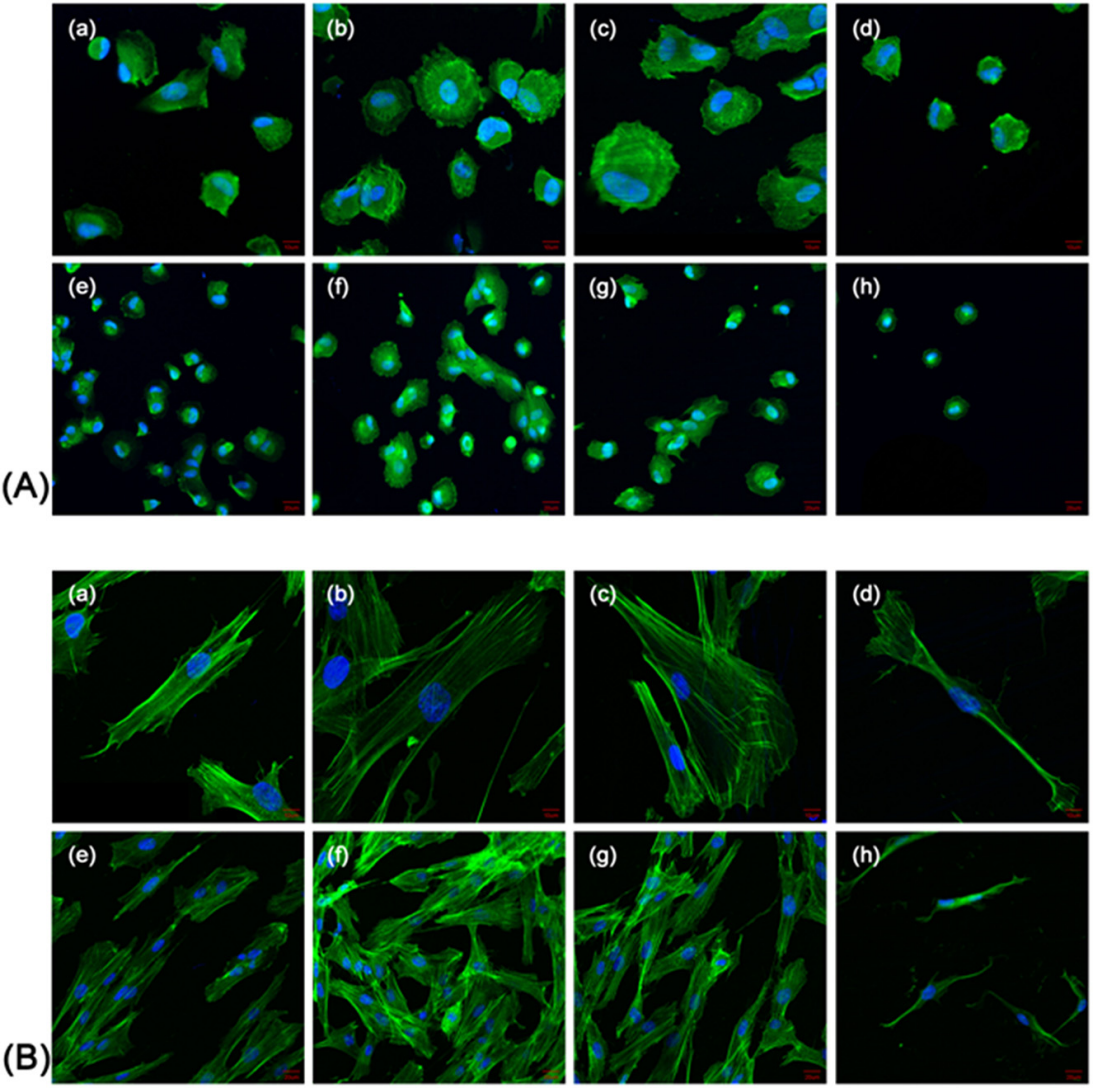
Confocal laser scanning microscopy observations of the HGFs on the control and plasma treatment surfaces. Confocal laser scanning microscopy images of HGFs on zirconia disks at 3 h (A) and 24 h (B) of culture. The helium plasma treatment time are 30 s (a, e), 60 s (b, f), 90 s (c, g), and 0 s as controls (d, h). High magnification (a-d), scale bar = 10 μm. Low magnification (e-h), scale bar = 20 μm.

**Fig 8:**
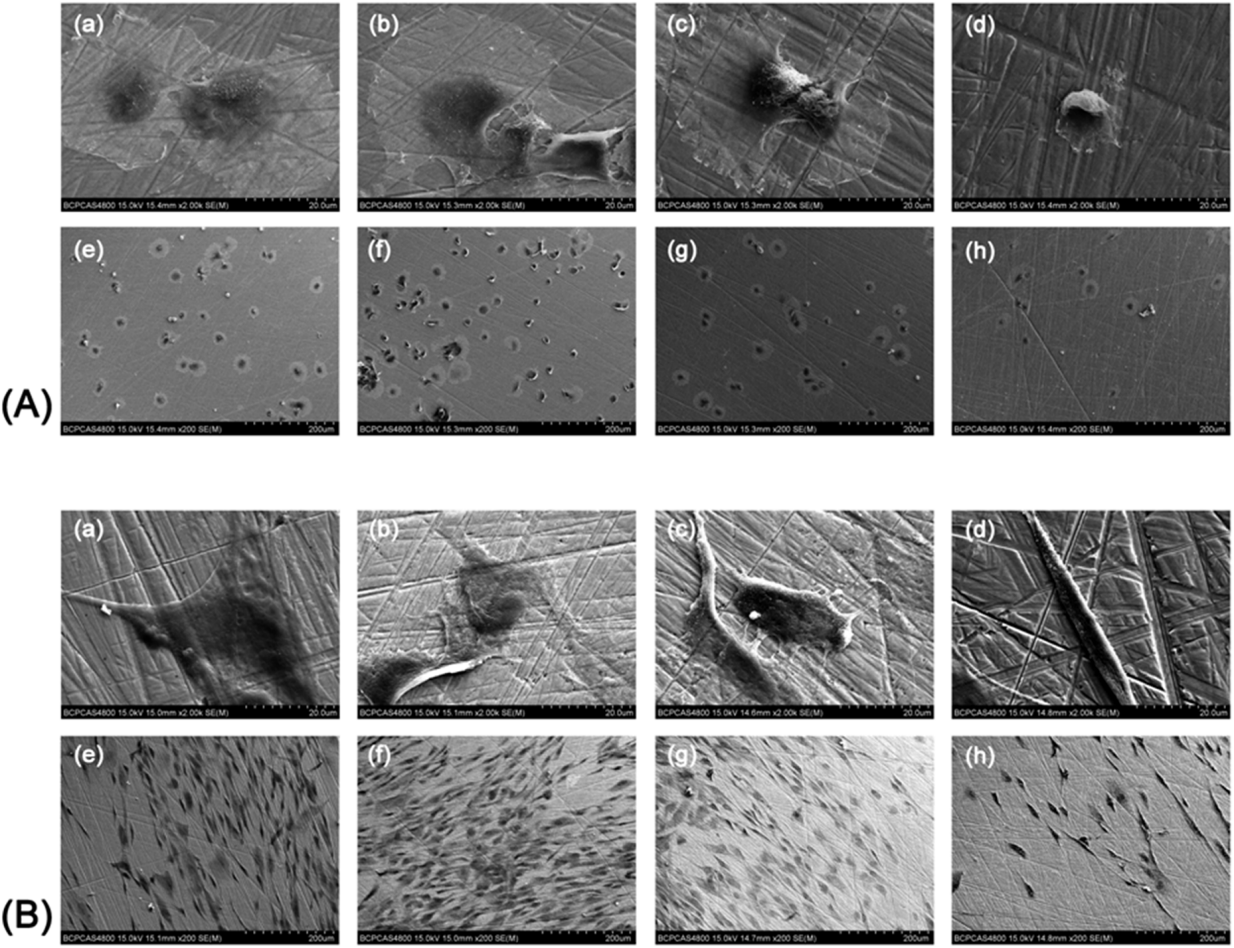
SEM observations of the HGFs on the control and plasma treatment surfaces. SEM images of HGFs on zirconia disks at 3 h (A) and 24 h (B) of culture. Helium APDBD plasma treated for 30 s (a, e). Helium APDBD plasma treated for 60 s (b, f). Helium APDBD plasma treated for 90 s (c, g). Surfaces without helium APDBD plasma treatment (d, h). High magnification (a-d), scale bar = 20 μm. Low magnification (e-h), scale bar = 200 μm.

**Fig 9:**
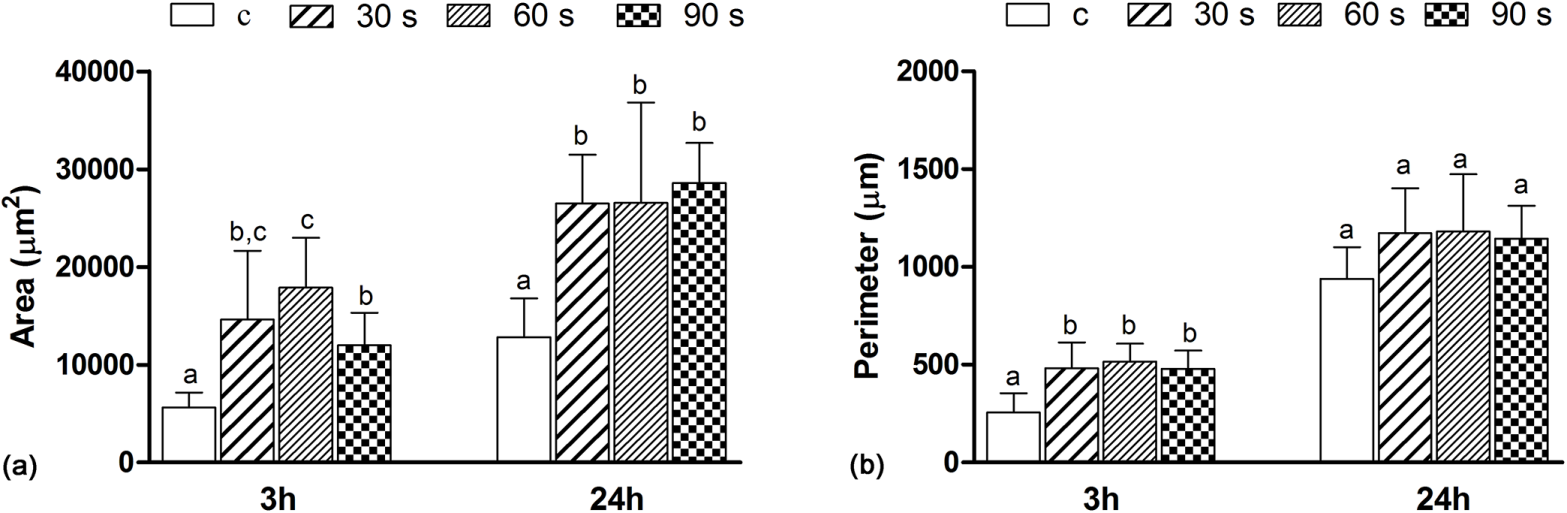
Spreading areas and perimeters of the HGFs on the control and plasma treatment surfaces. Spreading areas (a) and perimeters (b) of the HGFs on the control and plasma treatment surfaces at 3 h and 24 h. Data are shown as means ± SD (n = 15). Identical letters indicate no significant difference between those values (*p* > 0.05).

HGFs were attached on all surfaces after incubation for 3 h. Cells of the three helium APDBD plasma treatment groups spread better and had more protrusions, as well as larger surficial areas (Figs 7A and 8A). After 24 h of incubation, the shape of HGFs enlarged from round to microscler. Cells of the three treatment groups had more protrusions and intercellular interactions, while the cells of the control group had a spindle-like shape and narrow morphology with little protrusions (Figs 7B and 8B). The surficial areas of each group are shown in Fig 9A, which indicated that the HGF surficial areas were doubled, even tripled, in the treatment groups (30 s, 60 s) when cultured for 3 and 24 h. Cell perimeters in the plasma treatment groups were also doubled at 3 h, while there was no difference between the plasma treatment and control group at 24 h (Fig 9B).

### Influence of different surfaces on functional gene expression in HGFs

**Fig 10:**
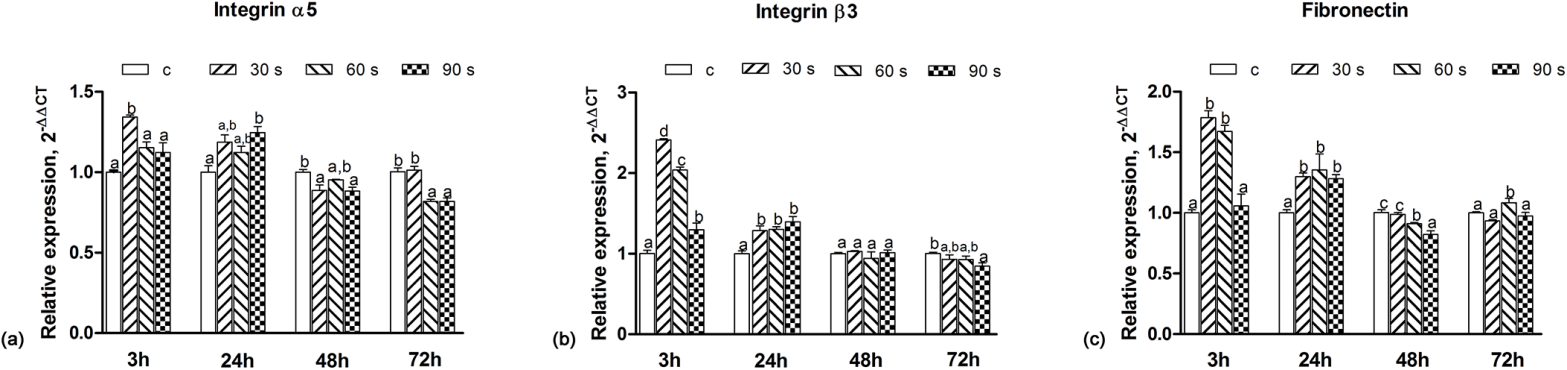
Analyses on the expressions of genes involved in the HGF adhesion. Analysis on the expression of integrin α5 (a), integrin β3 (b), and FN (c) after 3, 24, 48, and 72 h of culture in real-time PCR. Data represent fold changes of the target genes relative to the GAPDH expression and the HGFs grown in a control group (100%). The values are expressed as means ± SD. Identical letters indicate no significant difference between those values (*p* > 0.05).

The expression levels of HGF adhesion biomarkers on zirconia are shown in Fig 10. The expression levels of integrin α5 (30 s), integrin β3 (30 s, 60 s, 90 s) and FN (30 s, 60 s) at 3 h and integrin α5 (90 s), integrin β3 (30 s, 60 s, 90 s) and FN (30 s, 60 s, 90 s) at 24 h were significantly higher (*p*<0.05) than those of the control group; while with a further increase of the incubation time, the helium APDBD plasma treatment had no effects on improving the expression of the examined biomarkers. Among the groups treated by the helium APDBD plasma, the 30 and 60 s groups showed the most improvement in the expression of integrin β3 and FN at 3 h.

## Discussion

Due to its superior properties, the application of zirconia as an abutment material in the esthetic zone is becoming more and more popular. Yet implants in the anterior region have a higher requirement with regard to the shape and quality of peri-implant soft tissues. Since it was reported that the smooth zirconia surface has positive effect on the cells attachment and proliferation, e.g., with a 0.04-μm-in-roughness zirconia promoted the growth of HGFs [8,23], the zirconia surface with a roughness of 0.05 μm, which was below the threshold of bacterial aggregation [32], was chosen in this study.

As is known, many physical or chemical materials surface modification methods may change the tomography of the materials [25,28], and therefore, it would be difficult to distinguish which property of the material surfaces affects the behavior of the cells. In addition, some studies found that changing the tomography influences the mechanical properties of zirconia [10,33]. It is found that the wear debris of zirconia [34] and powders of PSZ (CaO-ZrO_2_) [35] might be toxic to hard and soft tissues. Based on the results of this study after being treated by the helium APDBD plasmas for 30, 60 and 90 s, the surface wettability increased and the elements changed significantly while with no changes in the tomography of the materials, which is consistent with previous studies using the oxygen plasmas at a low pressure (1.5 Pa) [28,36]. And Shon et al. also concluded that the plasma treatment could increase the wettability of the zirconia regardless of the influence of the surface tomography by using a pencil-type APDBD plasma jet using the helium-water vapor mixture as the plasma working gas [37]. Sabri et al. also confirmed that the argon plasma treatment had no physical damage to the polymer surface [38]. This allowed us to evaluate the effects of wettability on HGFs without changing the surface tomography of the materials.

Based on the results of superficial element analysis of different groups, the peak of hydroxide with a bonding energy of 532.5 eV existed in the three plasma treatment groups. The appearance of hydroxide might explain the increase of wettability after plasma treatment. The roles of this basic hydroxyl OH (b) groups on the osteoblast-titanium interactions have been proved [39]. So we could speculate that the hydroxide produced by the plasma treatment might enhance the biological behaviors of the HGFs on the zirconia disks.

The APDBD plasmas used in this study has the following advantages for the treatment of material surfaces in dentistry: similar gas temperature level to that of the intraoral environment, fast treatment speed and environmentally friendly. In addition, it also sterilizes the material surfaces simultaneously during the plasma treatment process. On the other hand, compared with plasma materials modification methods using low-pressure glow discharge, the unique features of the APDBD plasmas lie in the low capital costs and flexible operations due to the removal of the expensive and complicated vacuum system. Finally, it should be pointed out that helium was chosen to produce the cold plasmas in this study, since it is much easier to produce the stable, mild and uniform glow discharges at atmospheric pressure under lower breakdown/discharge voltages compared with those using other gases, such as argon, oxygen, nitrogen or air. However, helium is very expensive and becoming scarce. So, it is necessary to produce stable glow discharge plasmas using cheaper gases, e.g., nitrogen, oxygen or air, for promoting their actual clinical applications in future studies.

Increased wettability induced by plasma treatment could greatly promote the biological response of HGFs. The HGFs play an important role in the formation of the soft tissue seal between the abutment surface and surrounding gingival tissue [40]. Once seeded onto a biomaterial surface, cells undergo a series of events: attachment, spreading, cytoskeletal development and formation of cell-matrix adhesions. Argon plasma with oxygen admixtures was shown to enhance osteoblast size [29]. The morphological and morphometric analyses in this study showed that HGFs displayed larger superficial areas and perimeters on the zirconia surfaces treated by the helium cold plasmas at 3 h, which is consistent with previous studies. While as the culturing time increased, the slender shape of the HGFs in the control group resulted in lager perimeters but smaller areas.

The remarkable changes in hydrophilicity also have a significant influence on the HGF numbers. Previous studies have shown that the osteoblast behavior was improved on the zirconia surface treated by oxygen plasmas [36]. This study also demonstrated that the cell density in the helium APDBD plasma treatment groups was increased significantly.

As the initial step, attachment plays a considerable impact on the subsequent reactions [41]. Previous studies emphasized the roles of integrins in the integrin-mediated cell spreading since they are involved in the physical anchoring processes and in the signal transduction via the cell membrane [42,43], and they were also confirmed to play important roles in cell adhesion [44]. In this paper integrins α5 and β3 were evaluated because their functions in HGFs have been confirmed by previous studies [44,45]. Science FN is a protein involved in cellular attachment, and the increased expression of FN would lead to an increased cellular attachment [46], FN has become an important marker in the peri-implant soft tissue [47]. In this study, the expression levels of integrins α5 and β3 and FN were significantly higher in all the plasma treatment groups after culturing for 3 h, and the 30 and 60 s groups showed better performances than those of the 90 s group; while with a further increase of the culturing time, the effects on integrins and FN expression decreased gradually. On the other hand, when the culturing time was extended to 2 or 3 days, the expression of integrins and FN showed no advantages in the plasma treatment groups. This suggests that the helium APDBD plasma has an early and short-term effect on the expression of integrins and FN, and that this effect lasts for approximately 1 day. This is consistent with previous studies showing that the cold plasma treatment has a short-term effect lasting no longer than 48 h [48].

Different cold plasma treatment times can induce different degrees of surface wettability [49]. Among the three plasma treatment groups, although the hydrophilicity increased with treatment time, the cells did not show correspondingly improved behavior. The preceding experimental results in this paper indicated that the cell density was maximal in the 60 s group at 3, 24, 48 and 72 h; and the 30 and 60 s groups showed significantly higher expression of integrins α5, β3 and FN at 3 h. This indicates that the biological behavior of the HGFs is optimal at a specific contact angle when other surface characters (e.g., roughness and chemical composition of the original substrate) remain the same, for example, an optimized adhesion and proliferation ability of the HGFs were obtained with a contact angle of approximately 50° in this study on the zirconia disk with an average roughness of 0.05±0.01 μm. This phenomenon may be explained as follows: On one hand, the longer plasma treatment time can give rise to an extensive bond breaking on the treated materials surface leading to the formation of low molecular weight oxidized carbon fragments which can, of course, gives rise to a more wettable surface; while on the other hand, these low molecular weight fragments are soluble in the culture medium. Since the disks were washed in PBS for cleaning in this study, this would lead to the non-monotonous variation of the biological behaviors of the HGFs with the increase of the plasma treatment time.

In a summary, the foregoing results show that high expression of integrins, particularly β3, promote the early formation of a FN matrix, which provides binding sites for regulatory factors and structural support for the cell adhesion, proliferation and spreading.

These results suggest that the helium APDBD plasma could enhance the behavior of HGFs, under a certain contact angle of zirconia disk, instead of the wettest surface.

## Conclusions

In this study, a simple, fast and effective way was proposed for the surface modification of zirconia abutments. After the helium APDBD plasma treatment, the attachment and proliferation of the HGFs were significantly improved, especially in the 60 s group. The gene expression experiment revealed that cold plasma treatment had a positive effect on the expression of attachment-related genes within 24 h, and the 30 and 60 s groups showed the best performance. The results of the morphological study were consistent with the gene expression analyses. This indicates that the helium APDBD plasma treatment is a potential technique with bright prospects for abutment surface modifications with improving the peri-implant soft tissue healing and to providing stable gingiva contours around the implant restorations in the esthetic zone.

## Supporting Information

S1 Data Raw values of the included studies. Raw values from the water contact angle analysis, optical density analysis, morphology analysis, XPS analysis, and real-time PCR in the study.
(XLSX)

S1 Fig An experimental design table of the study. (PNG)
