# Supplementary figures and images for: Enhanced Biological Behavior of In Vitro Human Gingival Fibroblasts on Cold Plasma-Treated Zirconia
Source: PLoS One. 2015 Oct 13;10(10):e0140278. doi: 10.1371/journal.pone.0140278 (PMC4603669; doi:10.1371/journal.pone.0140278)

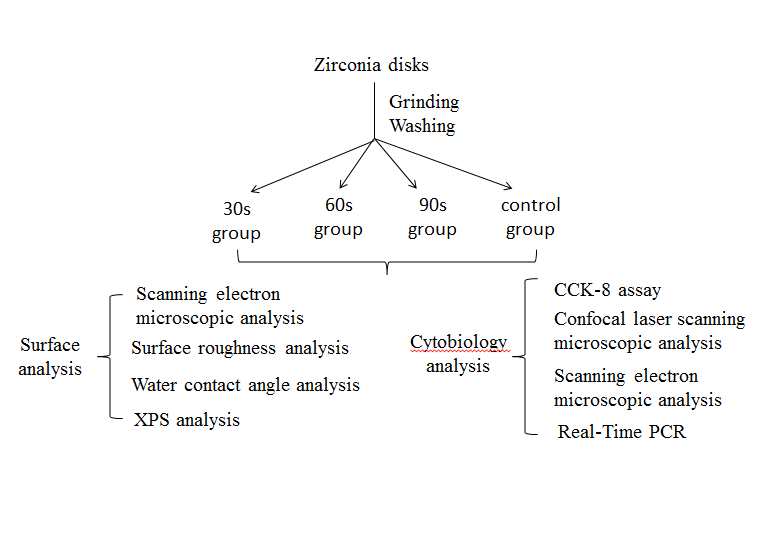

Supplement: S1 Fig — (PNG) [file pone.0140278.s002.png]
